# The role of BMP6 in the proliferation and differentiation of chicken cartilage cells

**DOI:** 10.1371/journal.pone.0204384

**Published:** 2019-07-01

**Authors:** Fei Ye, Hengyong Xu, Huadong Yin, Xiaoling Zhao, Diyan Li, Qing Zhu, Yan Wang

**Affiliations:** Institute of Animal Genetics and Breeding, Sichuan Agricultural University, Chengdu, China; Children's Hospital of Los Angeles, UNITED STATES

## Abstract

Previous studies have indicated that bone morphogenetic protein (BMP) 6 may play an important role in skeletal system development and progression. However, the mechanism underlying the effects of BMP6 in cartilage cell proliferation and differentiation remains unknown. In this study, cartilage cells were isolated from shanks of chicken embryos and treated with different concentrations of Growth Hormone. Cell proliferation potential was assessed using real-time polymerase chain reaction (RT-PCR), western blotting and CCK-8 assays in vitro. The results showed that at 48 h, the Collagen II and BMP6 expression levels in 50 ng/μl GH-treated cartilage cells were significantly higher than in groups treated with 100 ng/μl or 200 ng/μl GH. We further observed that knockdown of BMP6 in cartilage cells led to significantly decreased expression mRNAs and proteins of Collagen II and Collagen X. Moreover, the suppression of BMP6 expression by a specific siRNA led to significantly decreased expression mRNA levels of IGF1R, JAK2, PKC, PTH, IHH and PTHrP and decreased protein levels of PKC, IHH and PTHrP. Taken together, our data suggest that BMP6 may play a critical role in chicken cartilage cell proliferation and differentiation through the regulation of IGF1, JAK2, PKC, PTH, and IHH-PTHrP signaling pathways.

## Introduction

Bone morphogenetic proteins (BMPs) are secreted-type multifunctional proteins belonging to the transforming growth factor (TGF)-β superfamily. Many studies have reported that BMPs play very important roles in bone formation and cartilage induction in both vertebrates and invertebrates [1,2]; moreover, they are also considered crucial molecules involved in cell growth, differentiation, chemotaxis and apoptosis during embryonic development and postnatal tissue remodeling [3]. BMPs stimulate target cells mainly through their specific type I and type II receptors on the cell membrane. When signal transduction occurs, BMPs usually combine with the type II receptor, then activation of receptor type I [4,5]. BMPs first bind to the receptors on the membrane and transmit this signal through the Smads pathway to promote the differentiation of chondrocytes into the osteogenic lineage [6]. In addition to the Smads signaling pathway, other signaling pathways can also be transmitted from BMP family, such as mitogen-activated protein kinase (MAPK) pathways [7,8].

In the BMP family, BMP2, 4 and 6 are all thought to play the most important roles in skeletogenesis. Many studies have suggested that BMP2 is a pivotal signal for the regulation of osteoblastogenesis [9]. Mas et al [10] also showed that BMP2 promotes the expression of IHH in anterior hypertrophic chondrocytes and the proliferation of chondrocytes. BMP6 is mainly expressed in cartilaginous tissue, where it stimulates mesenchymal cell differentiation into chondrocytes and promote the synthesis of chondrocytes and articular cartilage-specific glycoproteins [11]. BMP6 can also induce the differentiation of MSCs into chondrocytes [12]. In BMPs, BMP6 is a strong factor for bone induction [13]. In addition to the differentiation of MSCs, chondrocytes can be derived from BMSCs, ADSCs and other stem cells induced by BMP6 [14–16]. These findings indicate that BMP6 is an important regulator of bone and cartilage cell proliferation and differentiation. However, the biological activity of BMP6 in cartilage cell proliferation and differentiation, as well as related signaling pathways, has remained unclear. Therefore, a further understanding of the molecular mechanism of BMP6 in cartilage is urgently needed.

GH is an important regulatory factor for longitudinal growth of the bone [17]. Local injection of GH can increase the number of cartilage cells in rats [18].In this study, we first extracted and cultured cartilage cells from different breeds of chickens, and we then investigated the expression of BMP6 and the changes in expression of key genes involved in related signaling pathways through GH (Growth hormone)-mediated induction at different concentrations to determine its potential role in cell proliferation and differentiation. Finally, to explore the mechanism of BMP6-mediated effects on the proliferation and differentiation of cartilage cells, we modulated the expression of BMP6 through siRNA and measured its effects by quantitative real-time PCR analysis. Collectively, this study provides evidence that within cartilage cells, BMP signaling regulates genes associated with both cell proliferation and differentiation.

## Materials and methods

### Animals

Avian broiler and Yellow bantam chickens, which have major differences, were used in this study. Avian broilers were provided by the Zheng Da Company (Chengdu, China). Yellow bantam chickens were provided by the Jin Ling Company (Guangzhou, China). All animal studies were performed in accordance with appropriate guidelines. All experimental protocols were approved by the Committee on Experimental Animal Management of Sichuan Agricultural University, permit number 2014–18.

### Cell culture

The eggs were incubated for 15 days after sterilization. Break the egg shell on the end of the air chamber with forceps and place the whole chicken embryo into the sterile culture dishes in PBS solution. Primary cartilage cells were isolated from the shank of 15-day-old chicken embryos with 0.25% trypsin (Gibco, USA), digestion for 0.5 h and then 0.1% collagenase Ⅱ (Gibco, USA) for 1.5 h at 37°C under sterile conditions. The cells were grown in DMEM/F12 medium (Gibco, USA) supplemented with 10% fetal bovine serum (FBS, Gibco, USA), 100 U/ml penicillin and 100 U/ml streptomycin (Gibco, USA) in a humidified atmosphere of 5% CO_2_ at 37°C.

### Immunofluorescence

Cartilage cells were fixed in 4% paraformaldehyde for 20 min after adhering for 24 h in the incubator and were then washed three times in PBS, 5 min per wash. The cells were permeabilized with 0.5% Triton-X-100 (Gibco, USA) for 15 min at room temperature and then washed three times in PBS, 5 min per wash. The cells were blocked with Blocking buffer (Bio-Rad, USA) for 2 h at 37°C and then washed as above. The cells were incubated in PBS containing an antibody towards type II collagen (1:1000) (Abcam, USA) overnight at 4°C. The following day, the cells were washed as described above and incubated in PBS containing IgG (1:250) (Abcam, USA) for 2 h at 37°C under dark conditions. After washing three times, the cells were counterstained with DAPI. Photomicrographs were taken using an Olympus digital camera system.

### Cell viability assay

Cell proliferation activity was evaluated via Cell Counting Kit-8 assay (CCK8, Bioss, China). At different time points, the medium was replaced with 100 μl of fresh medium containing 10 μl of CCK8. Three hours after the addition of CCK8, cell viability was determined with a microplate reader (Thermo Electron, USA) at a wavelength of 450 nm. All plates had control wells containing medium without cells to obtain a value for background luminescence, which was subtracted from the test sample readings. Each experiment was performed in triplicate. The growth kinetics were performed by the OD values (y axis) and day times (x axis).

### Cell treatments

Cells were treated with 0 ng/μl, 50 ng/μl, 100 ng/μl, and 200 ng/μl GH (Biovision, USA) while cells were grown to 80% confluence. Cells were harvested after transfection for 24 h, 48 h, and 72 h. Each experiment was performed in triplicate.

Two siRNAs were designed based on the sequence from NCBI (XM-418956.4) and are listed in Table 1. The cartilage cells were grown to 80% confluence and transfected with 5 μl of Lipofectamine 2000 (Invitrogen, USA) and 5 μl of siRNA (Sangon Biotech, China) according to the manufacturer’s instructions. Cells were harvested after transfection at 24 h, 48 h, and 72 h. Each experiment was performed in triplicate.

**Table 1:** Sequences of siRNA targeting the BMP6 gene.

siRNA		Sequences (5’-3’)
siBMP6.1	sense	CCCAATGACACCAAATCAATT
	antisense	TTGATTTGGTGTCATTGGGTT
siBMP6.2	sense	GCGAACAGCTCATCTGTGTTT
	antisense	ACACAGATGAGCTGTTCGCTT

### RNA extraction and qRT-PCR

Total RNA was extracted from cells using the RNAiso plus Reagent (Takara, Japan) according to the manufacturer’s instructions. The first-strand cDNA was synthesized using the PrimeScript RT reagent Kit reverse transcriptase (Takara, Japan) with 1 μg of total RNA according to the manufacturer’s instructions. β-actin was chosen as an internal standard to control for variability in amplification due to differences in the starting mRNA concentrations. The forward and reverse primer sequences used to amplify the genes were designed according to the sequence retrieved from the NCBI and listed in Table 2. Quantitative PCR was performed using SYBR Green PCR technology with a Bio-Rad CFX Connect Real Time System (Bio-Rad, USA). PCR was performed at 98°C for 120 s, followed by 40 amplification cycles (98°C for 2 s, X°C for 15 s, 72°C for 10 s), followed by 65°C to 95°C per second and then 4°C forever. The relative gene expression level was calculated using the 2^− ΔΔC^_T_ method. All PCR runs were performed in triplicate.

**Table 2:** Primer sequences for qRT-PCR.

gene	primer	Sequence (5’-3’)	Temperature
*BMP6*	F	CACGCCATCGTCCAAACTCT	60
	R	TGACATCCACAGGCTCTTACTACC	60
*β-actin*	F	TGTGCTGTCCCTGTATGCCTC	60
	R	GGAGGGCGTAGCCTTCATAGA	60
*BMP2*	F	TGGTGGAGGTGGTTCACTTGGA	60
	R	TGTTTGTGTTTCGCTTGACGCTTT	60
*Collagen II*	F	CCACCCTCAAATCCCTCAACA	60
	R	GTAATCTCCGCTCTTCCACTCG	60
*Collagen X*	F	GCCCAGGGTAACAGGGGTCTTC	60
	R	GCTTGCCGATGCCAACTTCT	60
*IGF1R*	F	CTTTGTCCCCAGACCTGATCG	60
	R	GAACGGGTATTCCACCTCACTC	60
*IHH*	F	CGCTCGCCTACAAGCAGTTC	60
	R	GGTTGTAGTTGGGCGTGAGC	60
*JAK2*	F	AGTCTTGGGCAGGGCAGATT	60
	R	TTGCTGCCTCAAAGAAGGACTC	60
*PKC*	F	GCTGACGAGGATGGAAATGC	60
	R	TTCACCCTGTCCAGATTGTTG	60
*PTHrP*	F	GTTATTCTGTGCCCTCCTACGG	60
	R	TCTTGGATTGATTTGCCCTTG	60
*PTH*	F	ACAGTGCCCTTGAGGATGCC	60
	R	CAATGGATTTCTTCTGCACTGC	60

### Western blotting

Proteins were extracted from cells by total proteins extraction kit (Tiangen, China) according the manuscripture. Total proteins were separated on a 12% SDS-PAGE, and transferred to a polyvinylidene fluoride membrane (Beyotime, China). The membrane was blocked in 5% BSA blocking solution for 1 h at room temperature and incubated overnight at 4°C with primary antibodies (Bioss, China) as follows: BMP6 (bs-10090R, diluted 1:500), Collagen II (bs-10589R, diluted 1:500), Collagen X (bs-0554R, diluted 1:500), BMP2 (bs-10696R, diluted 1:500), JAK2 (bs-0908R, diluted 1:500), PKC (bs-3729R, diluted 1:500), IHH (bs-6624R, diluted 1:500), PTHrP (bs-1107R, diluted 1:500), IGF1R (bs-0680R, diluted 1:500) and beta-actin (bsm-33036M, diluted 1:1,000). After that, the membrane was washed with scrubbing solution (Beyotime, China) and then developed with anti-mouse or rabbit horseradish peroxidase conjugated secondary antibodies (Beyotime, China, diluted 1:1,000). Protein bands were visualized using enhanced chemiluminescence (ECL) system (Beyotime, China) and quantified with an Image Lab system (Bio-Rad, USA).

### Statistical analyses

All cell culture experiments were performed a minimum of three times. Statistical analyses were conducted using SAS 8.0 software for Windows. All data are expressed as means ± SEM, and statistical analysis was performed usingone-way ANOVA. P-values < 0.05 were considered statistically significant.

## Results

### Immunofluorescence of cartilage cells

**Fig 1:**
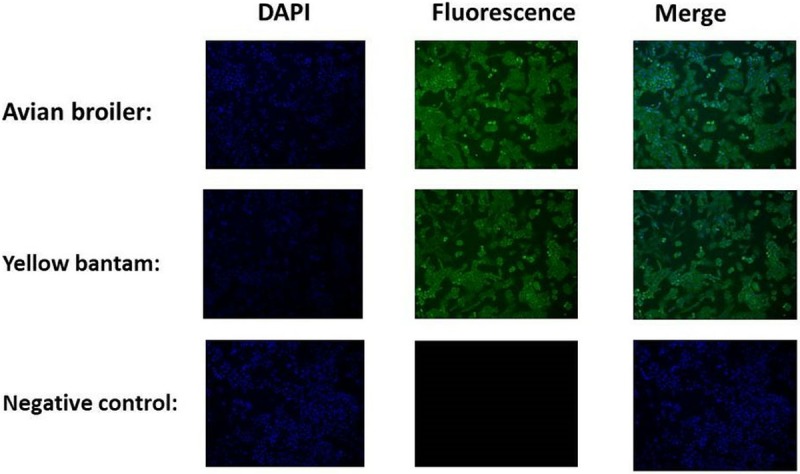
Immunofluorescence of markers in cartilage cells. Nuclei stained with DAPI are shown in the left panels. The pictures above indicated that staining of the cells for the marker collagen II was positive. The merged images are shown in the right-most panels. Scale bar = 100 μm.

Collagen II as a special marker for cartilage cells [19–26] was imaged in cartilage cells isolated from avian broiler and yellow bantam chickens, and the cells morphologies of the two breeds was consistent (Fig 1). These results indicate that the cells we cultured were cartilage cells.

### Growth kinetics of cartilage cells of the two breeds

**Fig 2:**
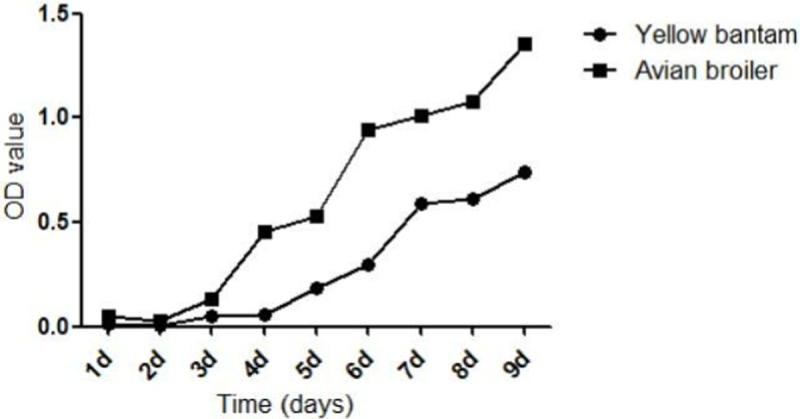
Growth curves of two breeds cartilage cells had no significant difference. The growth curves of cells were typically sigmoidal, with cell density reflected by the vertical axis. The growth curve consisted of a latent phase, a logarithmic phase, and a plateau phase (n = 3).

The growth kinetics of the cartilage cells from the two breeds at different time points are shown by the growth curves. Avian broiler cartilage cells entered the logarithmic phase after approximately 3 days, which ended at the ninth day, whereas yellow bantam cartilage cells entered the logarithmic after approximately 4 days and ended at the tenth day (Fig 2). Avian broiler cartilage cells might have stronger proliferation than yellow bantam cartilage cells, but there were no significant difference between the two breeds according the growth kinetics of cartilage cells.

### Expression of *BMP6* in cartilage cells of the two chicken breeds

**Fig 3:**
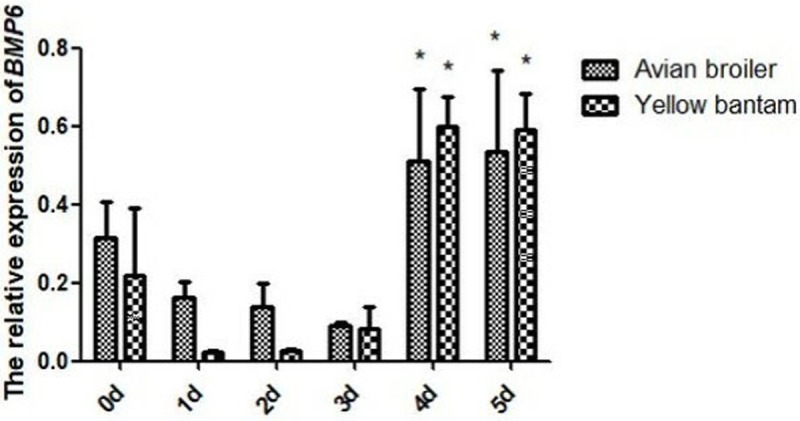
The expression of *BMP6* gene was consistent with the growth curves at different days. All values are presented as the means ± SEM (n = 3). (*) represents statistical significance (P < 0.05).

The relative expression level of *BMP6* mRNA was detected in cartilage cells from avian broilers and yellow bantams at day 0, day 1, day 2, day 3, day 4, and day 5 according to the growth curves of cells (Fig 3). Real-time PCR experiments showed that the cellular expression of *BMP6* significantly (P < 0.05) increased at the fourth day and fifth day, which was consistent with the growth curves.

### Expression of *BMP6* mRNA after GH induction

**Fig 4:**
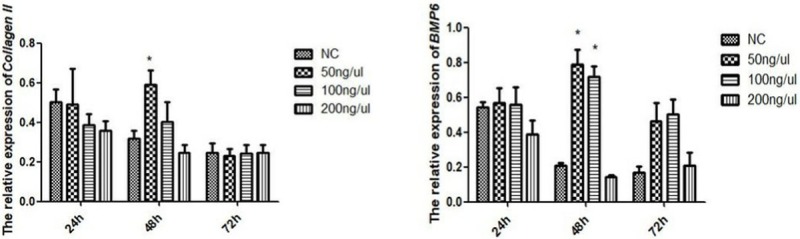
The relative expression of *Collagen II* and *BMP6* mRNA in cartilage cells after GH induction was significantly increased. All values are represented as the means ± SEM (n = 3). (*) represent statistical significance (P < 0.05).

**Fig 5:**
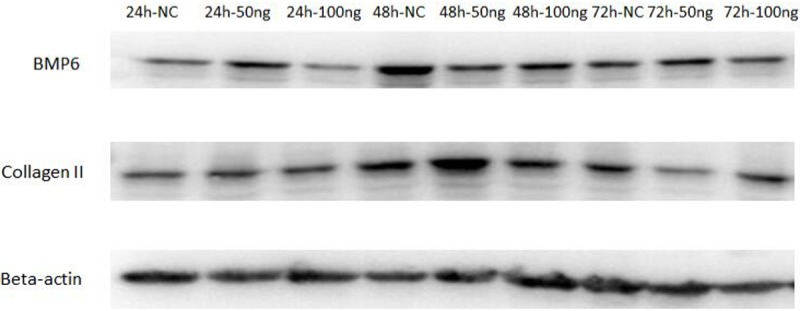
**The protein levels of Collagen II (A) and BMP6 (B) by Western blot in cartilage cells after GH induction was significantly increased.** The protein beta-actin was used as reference gene.

Based on the growth curves and the relative expression of *BMP6* mRNA in cartilage cells of avian broilers and yellow bantams, we selected cartilage cells of avian broilers for induction by GH and interference by siRNA targeting BMP6. Collagen II, a special marker for cartilage cells, was detected in cartilage cells after GH-induction. Relative to the β-actin gene, the expression levels of *BMP6* mRNA varied considerably at different times (Fig 4). The expression levels of *Collagen II* and *BMP6* in the three treatment groups at 24 h and 72 h was not significantly (P > 0.05) higher than that in the blank treatment group. However, at 48 h, the proliferation of cartilage cells was significantly (P < 0.05) elevated in the treatment group 50 ng/ul compared to that of the blank group, 100 ng/μl and 200 ng/μl groups (Fig 4). Considering the proliferation of cartilage cells of 200 ng/ul treatment group wasn’t significantly (P > 0.05) changed at 24 h, 48 h and 72 h, we detected the proteins of Collagen II and BMP6 in the blank group, 50 ng/ul and 100ng/ul treatment groups. The protein of Collagen II at 48 h in the 50 ng/ul treatment groups was significantly (P<0.05) higher than the blank group and 100 ng/μl groups (Fig 5). These results indicated that the cells were most sensitive to the concentration of 50 ng/μl GH. The relative expression levels of *BMP6* mRNA in cartilage cells after induction are shown in Fig 4 (B). In the 50 ng/μl and 100 ng/μl treatment groups, the expression of *BMP6* mRNA was significantly (P < 0.05) increased relative to the blank treatment group and 200 ng/μl at 48 h, which was consistent with the expression of *Collagen II* mRNA. However, the protein of BMP6 showed no significantly changes between treatment groups and blank group (Fig 5). This showed that there might be post-transcriptional modification to regulate the expression of *BMP6* in the process of proliferation of cartilage cells.

### The interference efficiency of the two siRNAs

**Fig 6:**
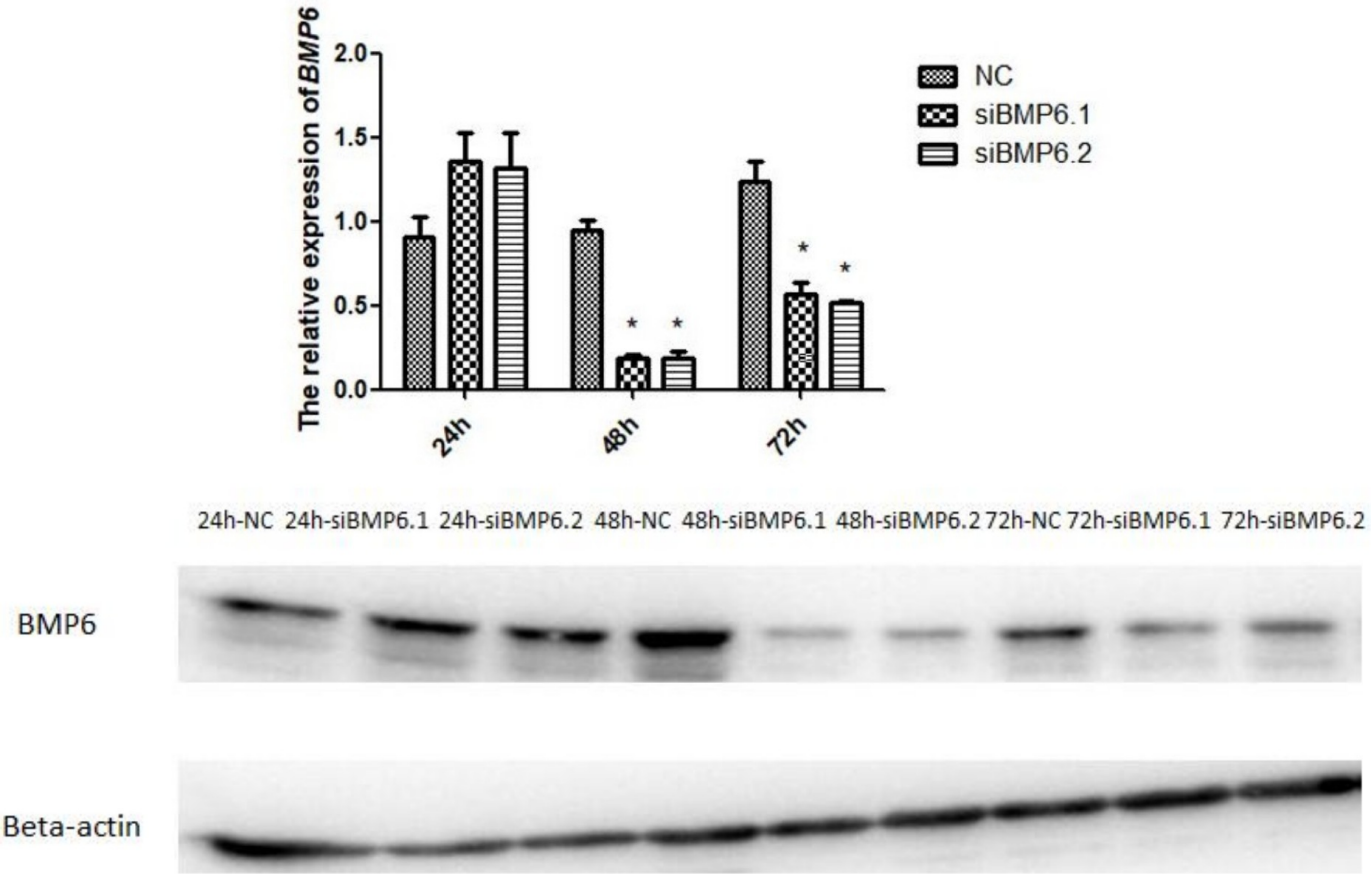
The interference efficiency of the two siRNAs were marked and stable. **(A)** The expression levels of ***BMP*** mRNA by qRT-PCR. All values represent means ± SEM (n = 3). (*) represents statistical significance (P < 0.05). (B) The expression levels of **BMP6** protein by western blot. The protein beta-actin was used as reference gene.

It can be seen from Fig 6 that the relative expression of *BMP6* mRNA and protein levels of cartilage cells after interference with siBMP6.1 and siBMP6.2 at 24 h was not significantly (P > 0.05) different from the control group, indicating that the efficiency of siRNA interference was not reflected at this time. At 48 h and 72 h, the mRNA and protein levels of *BMP6* in the treatment groups were significantly (P < 0.05) decreased compared with the control group. The interference efficiency of the two siRNAs at 48 h and 72 h were marked and stable, indicating their utility for subsequent experiments.

### The expression levels of *Collagen II* and *Collagen X* after interference with *BMP6*

**Fig 7:**
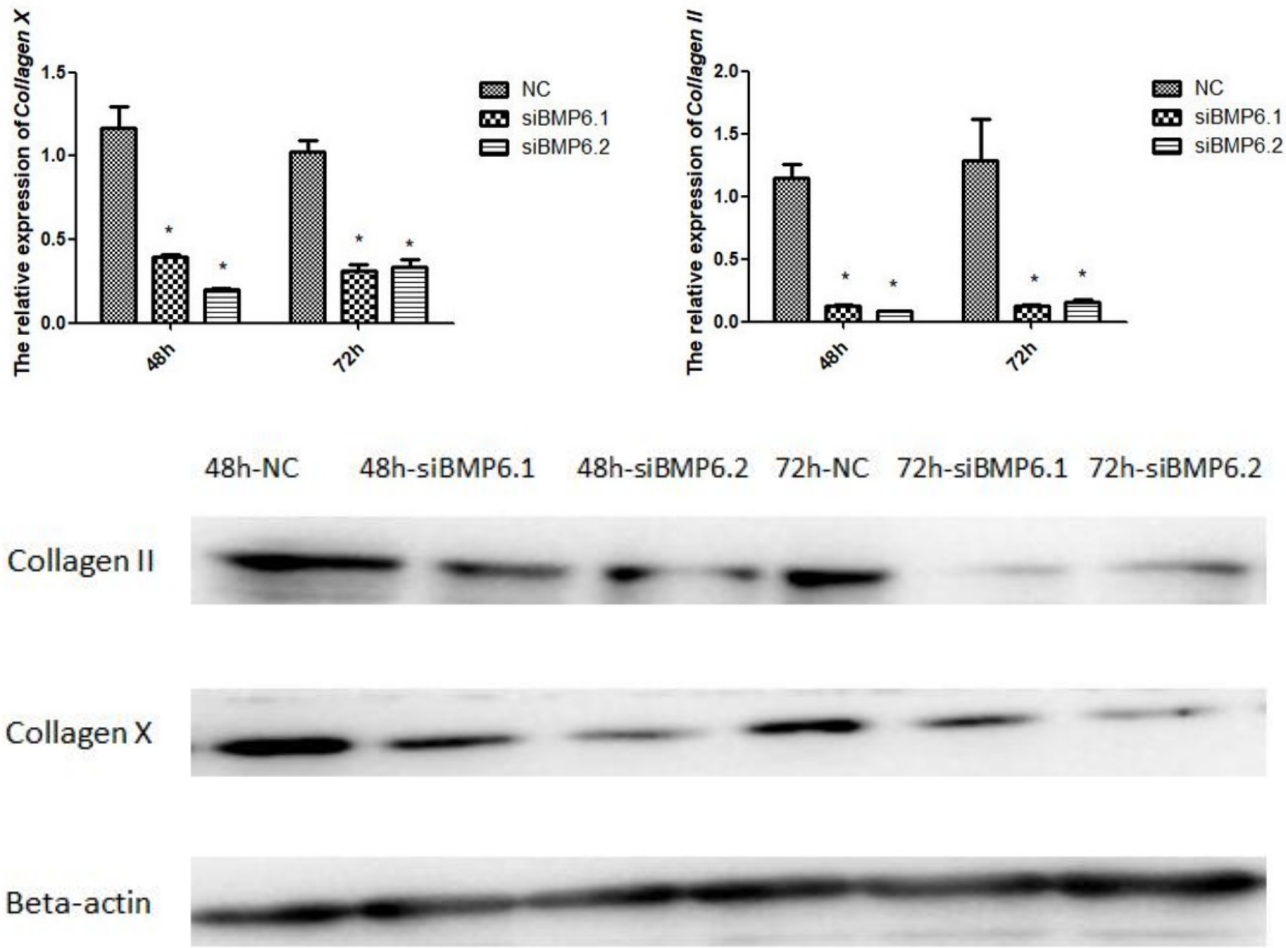
The expression of *Collagen II* and *Collagen X* in cartilage cells were significantly decreased after siRNA treatment. **(A)** The expression levels of ***Collagen II* and *Collagen X*** mRNA by qRT-PCR. All values are represented as the means ± SEM (n = 3). (*) represents statistical significance (P < 0.05). (B) The expression levels of **Collagen II and Collagen X** protein by western blot. The protein beta-actin was used as reference gene.

Collagen II and Collagen X have been suggested to be important markers of chondrocyte proliferation and differentiation [19–26]. We therefore further measured their gene expression levels and protein levels after interfering with *BMP6* expression. As shown in Fig 7, we found that at 48 h and 72 h after interference with *BMP6*, the *Collagen Ⅱ* and *Collagen X* mRNA expression and protein expression levels in chondrocytes were significantly (P < 0.05) lower than in the control group.

### The expression levels of *BMP2* after interference with *BMP6*

**Fig 8:**
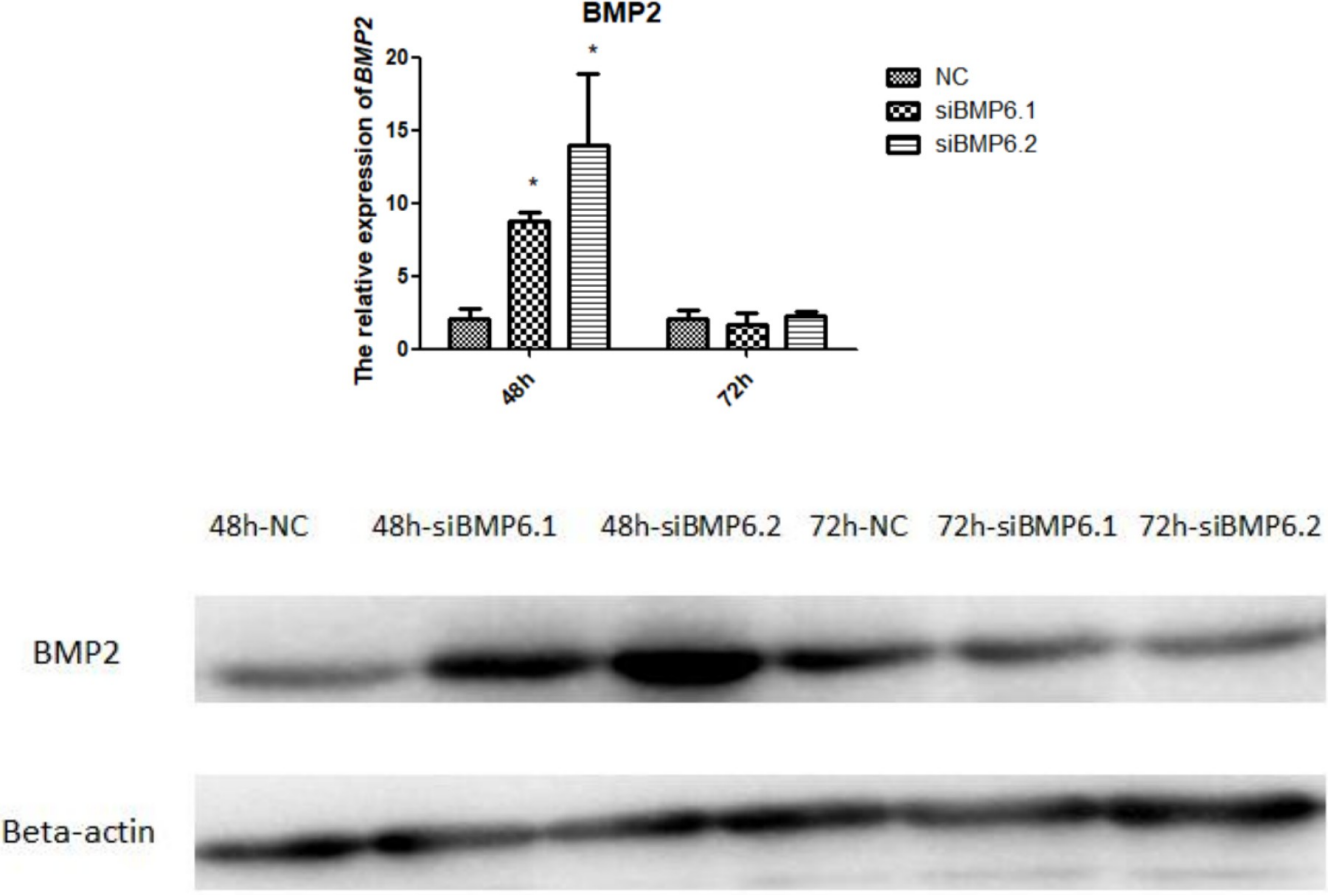
The expression of *BMP2* in cartilage cells were significantly increased at 48 hafter siRNA treatment. (A) The expression levels of *BMP2* mRNA by qRT-PCR. All values are represented as the means ± SEM (n = 3). (*) represents statistical significance (P < 0.05). (B) The expression levels of BMP2 protein by western blot. The protein beta-actin was used as reference gene.

We measured the *BMP2* expression levels after interfering with *BMP6* expression. As shown in Fig 8, we found that at 48 h after interference with *BMP6*, the *BMP2* mRNA expression and protein expression levels in chondrocytes were significantly higher than in the control group (P < 0.05), but there was no significant (P > 0.05)difference between treatment group and control group at 72 h.

### The expression levels of *IGF1R*, *JAK*, *PKC*, *PTH* and *PTHrP* after interference with *BMP6*

**Fig 9:**
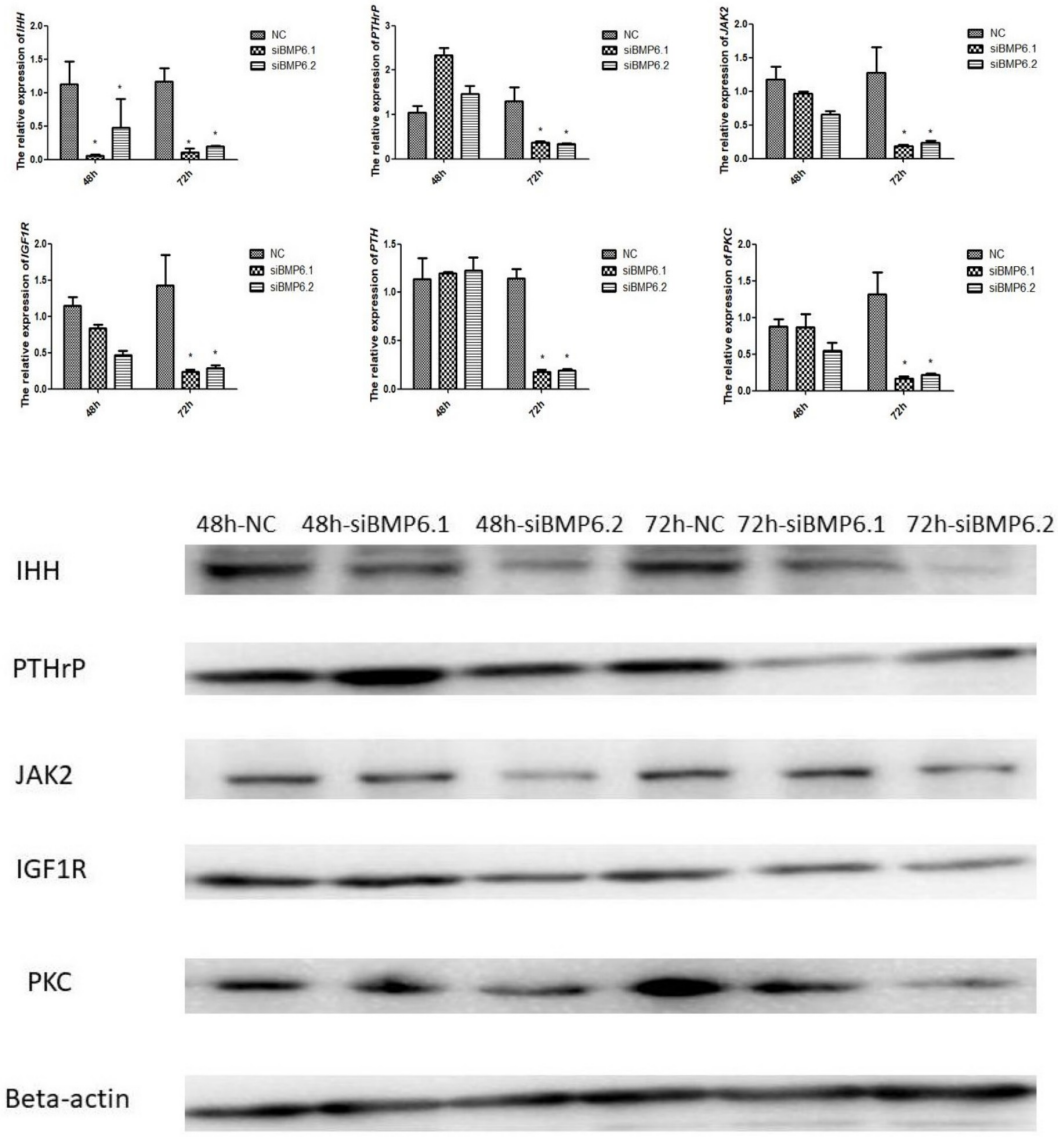
The mRNA expression of selected genes in cartilage cells after siRNA treatment were significantly decreased. **(A)** The expression levels of genes mRNA by qRT-PCR. All values represent means ± SEM (n = 3). (*) represents statistical significance (P < 0.05). (B) The expression levels of proteins by western blot. The protein beta-actin was used as reference gene.

Since IGF1, PKC, JAK2/STAT, IHH/PTHrP and PTH signaling have been reported to affect the proliferation and differentiation of chondrocytes, we further investigated whether BMP6 was also involved in these processes. We used siRNA to knock down BMP6 gene expression in plasmid-transfected cells. Real-time PCR analysis showed that transfecting chondrocytes with BMP6-targeting siRNA resulted in a significant inhibition of expression of *IHH*, *IGF1R*, *JAK2*, *PKC*, *PTH* and *PTHrP* at 72 h (Fig 9). However, at 48 h, the relative expression of *IGF1R*, *JAK2*, *PKC*, *PTH* and *PTHrP* mRNAs had no significant (P > 0.05) differences between the treatment and control cartilage cells, whereas the relative expression of *IHH* in the treatment groups was significantly (P < 0.05) lower than in the control group.

The proteins of IHH, PTHrP and PKC at 72 h in the treatment showed significantly (P < 0.05) lower than in the control group. The proteins of JAK2 and IGF1R had no significant (P > 0.05) differences between the treatment and control cartilage cells. As there wasn’t appropriate primary antibody for PTH, we didn’t detect the protein of PTH.

## Discussion

Currently, studies regarding the BMP6 gene in chondrocytes have primarily focused on humans, mice, rabbits and other mammals, with few studies focused on birds. Previous study demonstrated that BMP6 function in the appendicular bone development was dispensable but was required for the progression of ossification of the sternal elements [27]. Considering the biological difference between mammals and birds, there may be some different functions of BMP6. As members of BMPs, BMP2 and BMP4 induce an increase in the volume of skeletal elements by recruiting non-chondrogenic precursors to form cartilage [28,29]. But BMP4 is more effective than BMP2 at promoting differentiation [28]. BMP6 stimulates the phenotypic changes associated with chondrocyte differentiation such as increased alkaline phosphatase activity and decreased proliferation [29]. BMP6 has high levels of expression in the developing growth plate, which is on the contrary with BMP2, BMP4 and BMP7 that are expressed at lower levels or are absent in the growth plate[30,31]. In our previous studies we found that the polymorphism in BMP6 gene in chicken had significant correlation with femur perimeter [32], but the expression of BMP6 in cartilage plate of 15 E, 1 day avian broiler and yellow bantam chickens detected by RT-PCR was high (not published). BMP6 gene plays an important role in the chondrocytes in the chicken embryonic development. But the research about the signaling pathway of BMP6 is few except the SMADS signaling pathway. To figure out the signal pathways related to BMP6, we used immunofluorescence to identify and observe the cell morphology, used CCK8 assays to detect the proliferation of cartilage cells and real-time PCR to detect the *BMP6* mRNA expression in cartilage cells cultured from avian broiler and yellow bantam chickens. Collagen II is a representative protein indicating the proliferation of chondrocytes, and thus can be used as a cell marker; many studies have examined the expression of collagen II in the mandibular condyle, using both immunohistochemistry and in situ hybridization techniques [19–26]. Our results showed that the avian and yellow bantam cartilage cells had no significant difference. According to these results, we selected cartilage cells cultured from avian chicken growth plates to research the function of BMP6 for regulating the proliferation and differentiation of cartilage cells.

The expression of GH can promote the expression of *Collagen II* mRNA in cartilage cells and maintain a chondrocyte phenotype. We used GH to induce the cartilage cells in order to detect the expression of *BMP6* mRNA in proliferative cells. The results showed that the sensitivity of cartilage cells to GH varied with GH concentration. When cartilage cells proliferated, the expression of *BMP6* mRNA was significantly increased but the protein levels of BMP6 wasn’t significantly changed. Therefore, we speculated that BMP6 participates in the proliferation of cartilage cells or promote cells differentiation to relieve the competitive pressure brought by cell proliferation. But the proliferation of cartilage cells controls the expression of BMP6 to maintain the proliferation state.

RNA interference uses homologous double-stranded RNA (dsRNA) to induce the silencing of specific target genes and block gene activity. SiRNA (small interfering RNA) is the intermediate of RNA interference, which is necessary for the RNA interference and can stimulate the complementary target mRNA silencing. Collagen X is specifically expressed in hypertrophic chondrocytes [33]. In this experiment, two siRNAs were designed and synthesized according to the BMP6 gene CDS (NCBI, XM-418956.4) region, and the expression of BMP6 mRNA was inhibited. BMP6 was involved in the proliferation of cartilage cells, and, when the expression of BMP6 mRNA was suppressed in cartilage cells, the expression of *Collagen II* and *Collagen X* mRNA—two marker genes representing the proliferation and differentiation of cartilage cells—decreased in cartilage cells. These results showed that the proliferation and differentiation of chondrocytes were both blocked following the disruption of the expression of BMP6. Together with the previous results, we speculate that BMP6 is involved in the proliferation and differentiation of cartilage cells, which is consistent with the existing research findings. IGF1 plays an important role in the growth and development of bone cells; it can regulate the function of osteoblasts in various forms and participates in bone reconstruction. IGF1 can significantly promote the proliferation of bone progenitor cells both in vivo and in vitro [34]. The signal transmission mediated by IGF1 is specifically induced by the IGF1R on the cell surface, thus promoting the growth, differentiation and apoptosis of tissue cells. To explore whether IGF1R is involved in the regulation of chondrocyte proliferation and differentiation of BMP6, the expression of *IGF1R* mRNA and protein levels were detected while using RNAi technology to interfere with the expression of BMP6. When the expression of BMP6 was inhibited, the expression of *IGF1R* mRNA was also inhibited, otherwise the protein of IGF1R wasn’t significantly varied. Therefore, we speculate that BMP6 has a regulatory effect on IGF1R but there may be some post-transcriptional modification to regulate the protein level of IGF1R.

JAK2 is a common signaling pathway induced by multiple cytokine- and growth factor-mediated signaling within cells [35]. When JAK activity is inhibited, osteoarthritis articular chondrocytes can reproduce and differentiate normally [36]. Interleukin-6 and interleukin-7 can induce cartilage cell activity through the JAK2/STAT signaling pathway [37,38]. It has been found that BMP7 can promote osteogenic differentiation of osteoblasts through JAK2/STAT5B signaling [39], and we hypothesized that BMP6 may regulate the proliferation and differentiation of chondrocytes through the JAK2/STAT signaling pathway. To explore whether JAK2 is involved in the regulatory effects of BMP6 on the proliferation and differentiation of cartilage cells, we used RNAi technology to interfere with the expression of *BMP6* and subsequently detect the expression of *JAK2* in the JAK2/STAT signaling pathway. Our results showed that the expression of *JAK2* was also inhibited when the expression of *BMP6* was decreased, otherwise the protein of JAK2 wasn’t significantly varied. We hypothesize that BMP6 has regulatory effects on JAK2 expression but there may be some post-transcriptional modification to regulate the protein level of JAK2.

PKC is an important substance in cell signal transduction pathways and participates in the process of proliferation and differentiation of chondrocytes; it is one of the important signal transducers affecting the growth and development of cartilage and its eventual degeneration [40]. PKC signaling and multiple other signaling pathways participate in IGF1 induction of chondrocyte proliferation and differentiation [41]. Estrogen and vitamin D-mediated signaling depends on the PKCα pathway to regulate the activity of cartilage cells [42]. To explore whether PKC is involved in the BMP6-mediated regulation of the proliferation and differentiation of cartilage cells, we used RNAi technology to interfere with the expression of BMP6 and detect the subsequent expression of PKC. Our results showed that *PKC* expression was inhibited both mRNA and protein levels when the expression of BMP6 was inhibited. We hypothesize that PKC is involved in the regulation of chondrocyte proliferation and differentiation mediated by BMP6. But the work to study the regulation is according phosphorylation or other modifications still needed more.

The main function of PTH is to regulate Ca2^+^ and Phosphorous metabolism and promote bone absorption. PTH stimulates the expression of Collagen II in cartilage cells [43]. A previous study found that PTH interacts with the TGF-beta signaling pathway at the receptor level [44]. BMP6 belongs to the TGF-beta family, therefore we speculated that PTH may participate in the regulation of chondrocytes by BMP6. We used RNAi technology to interfere with the expression of *BMP6* and detected subsequent *PTH* expression. Our results showed that *PTH* mRNA expression was also inhibited when the expression of *BMP6* was inhibited. We hypothesize that PTH participates in the regulation of chondrocyte proliferation and differentiation by BMP6.

IHH/PTHrP signaling is important in bone development regulation, modulating the differentiation of chondrocytes and osteoblasts, maintaining the cartilage cell proliferation state [45] and determining the length of the growth plate cartilage by regulating the bone longitudinal growth rate [46,47]. PTHrP can inhibit osteoblast differentiation by down-regulating BMP2 expression [47]. We hypothesized that the IHH/PTHrP signaling pathway may be involved in the regulation by BMP6 of chondrocytes. We used RNAi technology to interfere with the expression of *BMP6* and detect the subsequent expression of *IHH* and *PTHrP*. Our results showed that the expression of *IHH* and *PTHrP* mRNA and protein levels were also inhibited when the expression of BMP6 was decreased. The IHH/PTHrP signaling pathway participates in the regulation by BMP6 of the proliferation and differentiation of cartilage cells. But the work to study the regulation is according phosphorylation or other modifications still needed more.

While the expression of BMP6 was decreased, the expression of BMP2 was increased, the BMP2 may brevity replace the function of BMP6. That may be the reason the expression of IGF1/JAK/PKC/PTH/IHH-PTHrP weren’t decreased at 48 h but significantly decreased at 72 h. BMP6 belongs to the TGFβ family, and current research indicates that the regulation of chondrocytes is mainly achieved through the Smad signaling pathway of TGFβ [48]. Smads may be required, but not sufficient for the differentiation of chondrogenic differentiation of ATDC5 cells [49]. Further studies are needed to identity the signaling pathways responsible for biological effects. BMP2, BMP9 and BMP6 belong to the BMP family. BMP2 can increase the proliferation of chondrocytes and the elongation of chondrocytes by inducing the expression of IHH [10]. BMP9 and GH can synergize the osteogenic differentiation of mesenchymal stem cells through the JAK/STAT/IGF1 signaling pathway. As one of the strongest members of the BMPs family, BMP6 may regulate the proliferation and differentiation of chondrocytes through other signaling pathways in addition to the Smad signaling pathway. Therefore, we analyzed the IGF1/JAK/PKC/PTH/IHH-PTHrP signaling pathways, which play important roles in the process of proliferation and differentiation of cartilage cells, and detected the expression changes (mRNA and protein levels) of several key genes (IGF1R, JAK2, PKC, PTH, IHH, PTHrP) in these signaling pathways when the expression of BMP6was inhibited. The results collectively showed that the expression of IHH, PTHrP and PKC genes decreased significantly on mRNA and protein levels and the expression of IGF1R and JAK2 mRNA were significantly decreased while the protein levels of IGF1R and JAK2 had no significantly varied which might because there were less p+ signal transmit to the intracellular by BMP6 or because the cell activity was affected caused by the decreased expression of BMP6. IGF1/JAK/PKC/PTH/IHH-PTHrP are involved in the regulation by BMP6 of the proliferation and differentiation of cartilage cells, but there should be more work to verify how the signal pathway and its phosphorylation transmit. The work to study the regulation is according phosphorylation or other modifications still needed more.

## Conclusion

There was no significant difference in the shape, proliferation and differentiation of cartilage cells cultured from different chicken breeds. BMP6 was highly expressed when cartilage cells proliferated. When the expression of BMP6 was decreased, the proliferation and differentiation of chondrocytes was blocked, indicating that BMP6 is involved in the proliferation and differentiation of cartilage cells. The expression levels of key genes involved in the IGF1R, JAK2, PKC, PTH, IHH and PTHrP signaling pathways were significantly lower in cartilage cells when the expression of BMP6 was decreased. These key genes involved in signaling pathways may related to the regulation by BMP6 of the proliferation and differentiation of cartilage cells, but how the signal such as phosphorylation or other protein modification transmit still need more work to verify.
